# Effectiveness, safety, and impact on quality of life of eribulin‐based therapy in heavily pretreated patients with metastatic breast cancer: A real‐world analysis

**DOI:** 10.1002/cam4.6301

**Published:** 2023-07-05

**Authors:** Xinyu Gui, Xu Liang, Huiping Li

**Affiliations:** ^1^ Key laboratory of Carcinogenesis and Translational Research (Ministry of Education/Beijing) Department of Breast Oncology, Peking University Cancer Hospital & Institute Beijing, China 100142 China

**Keywords:** eribulin, heavily pre‐treated, metastatic breast cancer, real world study

## Abstract

**Introduction:**

Eribulin is currently recommended for the treatment of patients with metastatic breast cancer (MBC) pre‐treated with taxanes and anthracyclines. The aim of the present study was to evaluate the effectiveness and safety of eribulin and its impact on health‐related quality of life in heavily pre‐treated patients with MBC.

**Methods:**

Data from MBC patients who had received eribulin‐based therapy at Beijing Cancer Hospital between January 2020 and July 2022 were analyzed retrospectively. Progression‐free survival (PFS), overall survival (OS), objective response rate (ORR), disease control rate (DCR), adverse effects (AEs) and health‐related quality of life (HRQoL) were assessed.

**Results:**

Data from 118 patients who had received eribulin to treat MBC were included. Median PFS was 4.2 months and median OS had not been reached. The ORR was 13.6% (16/118) and DCR was 75.4% (89/118). The median PFS in patients who received eribulin in second‐line (26/118), third‐line (29/118), or fourth‐line or later (63/118) was 4.5, 4.2, and 3.9 months, respectively. The median OS in patients who received eribulin in third‐ or later line (*n* = 92) was 14.1 months. Patients who received eribulin combination therapy had a significantly longer median PFS compared with those who received eribulin monotherapy (4.5 vs. 3.4 months, *p* = 0.007) and there was a trend towards a longer median OS (not reached vs. 12.1 months). The most common grade 3–4 adverse events were neutropenia (22.9%), leukocytopenia (13.6%) and asthenia/fatigue (8.5%), without significant differences in safety between eribulin monotherapy and combination therapy. Quality of life was similar in patients who received eribulin monotherapy and combination therapy, except for cognitive function and nausea and vomiting symptoms, which were better with combination therapy.

**Conclusions:**

The present study suggests that eribulin‐based therapy is an effective treatment option and well tolerated for heavily pre‐treated patients with MBC. Eribulin combination therapy might improve PFS and HRQoL compared with eribulin monotherapy.

## Abstract 2

The present study suggests that eribulin‐based therapy is an effective treatment option and well tolerated for heavily pre‐treated patients with MBC. Eribulin combination therapy might improve PFS and HRQoL compared with eribulin monotherapy.

## 1 INTRODUCTION

Chemotherapy is essential in the management of metastatic breast cancer (MBC), although novel chemotherapeutic drugs have demonstrated limited benefit in recent years.^1^, ^2^ It remains challenging to improve the progression free survival (PFS) and overall survival (OS) in patients with MBC, particularly in heavily pre‐treated patients (such as those pre‐treated with taxanes and anthracyclines).^3^, ^4^

In contrast to taxanes and vinca alkaloids, eribulin inhibits the growth phase of microtubules but not the shortening phase. It also causes tubulin sequestration into non‐productive aggregates, and has other important antitumor effects such as tumor vascular remodeling and reversal of the epithelial‐mesenchymal transition.^5^, ^6^, ^7^, ^8^

In Chinese and international guidelines, eribulin is recommended for patients with MBC who have received prior treatment with taxanes and anthracyclines. This recommendation is based on several large, randomized, phase III clinical trials, including EMBRACE, the 301 and 304 studies.^9^, ^10^, ^11^ In the 301 study, the improvement in OS in MBC patients receiving eribulin compared with capecitabine was not statistically significant.^9^ In EMBRACE, eribulin improved OS in heavily pre‐treated patients with MBC versus with physician's choice of treatment, with manageable adverse events (AEs).^10^ In the 304 study, eribulin was associated with significantly superior PFS and tumor response rates compared with vinorelbine in Chinese patients with pre‐treated MBC.^11^

Patients with MBC often experience systemic symptoms and functional problems related to MBC itself or due to treatment that can affect health‐related quality of life (HRQoL) negatively.^12^, ^13^, ^14^ In phase III clinical studies, eribulin has been associated with AEs, most commonly asthenia/fatigue and neutropenia, and improved HRQoL, in comparision to other anti‐microtubule drugs.^9^, ^10^, ^15^ However, there are limited data regarding the use of eribulin on HRQoL in Chinese patients with MBC in real‐world settings.

Eribulin is used both as monotherapy and in combination with other anti‐tumor agents for the management of patients with MBC pre‐treated with anthracyclines and taxanes. Although previous studies have evaluated the use of eribulin in Chinese patients with MBC in real‐world settings,^16^, ^17^ these studies did not include data on HRQoL, which is highly important to support clinical decision making. Therefore, the present real‐world clinical study was designed to investigate the effectiveness and safety of eribulin, and also its impact on patients' HRQoL, given as monotherapy and combination therapy, in heavily pre‐treated Chinese patients with MBC.

## 2 METHODS

The medical records of adults with MBC who had received eribulin at Beijing Cancer Hospital between January 2020 and July 2022 were analyzed retrospectively.

### 2.1 Patients

Adults (18–80 years) with recurrent or metastatic breast cancer after surgery, or with initial stage IV inoperable breast cancer at diagnosis (confirmed by pathologic biopsy and immunohistochemical staining) who had received prior treatment with both taxanes and anthracyclines were eligible for inclusion if they had ≥1 measurable lesion according to the Response Evaluation Criteria in Solid Tumors (RECIST) version 1.1.^18^ Positivity for human epidermal growth factor receptor‐2 (HER2) was defined as immunohistochemical staining of 3+ or 2+ with gene amplification in fluorescence in situ hybridization in the primary and/or metastatic lesion, while hormone receptor (HR) positive was defined as estrogen and/or progesterone receptor ≥1% in primary and/or metastatic lesion. Patients were also required to have an Eastern Cooperative Oncology Group (ECOG) performance status of ≤2 and >3 months life expectancy.

### 2.2 Treatment

Eribulin was applied either as monotherapy or combined with other anti‐tumor therapy. Patients received eribulin 1.4 mg/m^2^ intravenously on day 1 and day 8 of each 21‐day cycle, with dose reductions and interruptions permitted in the event of eribulin‐related toxic effects, until disease progression or intolerable adverse effects. Eribulin was used in combination with anti‐angiogenic drugs (bevacizumab, anlotinib, and apatinib) and anti‐HER2 drugs (trastuzumab with or without pertuzumab, pyrotinib and neratinib). Patients received bevacizumab 7.5 mg/kg intravenously on day 1 of each 21‐day cycle, anlotinib 12 mg orally once on day 1 to day 14 of each 21‐day cycle, apatinib 500 mg orally once, trastuzumab 8 mg/kg (first cycle), 6 mg/kg (follow up) intravenously on day 1 of each 21‐day cycle, pertuzumab 840 mg (first cycle), 420 mg (follow up) intravenously on day 1 of each 21‐day cycle, pyrotinib 400 mg orally once and neratinib 240 mg orally once.

### 2.3 Evaluation of effectiveness

Tumor response was assessed using computed tomography (CT) or magnetic resonance imaging (MRI) based on RECIST version 1.1. Tumor responses included complete response (CR), partial response (PR), stable disease (SD), and progressive disease (PD). The proportion of patients with CR and PR defined the objective response rate (ORR), while the proportion of patients with CR, PR, and SD defined the disease control rate (DCR). PFS was defined as the time from initiation of eribulin until disease progression or death (in the absence of disease progression). OS was defined as the time from the initiation of eribulin until death from any cause. Patients were followed at every two therapy cycles routinely, including regular physical examinations, laboratory assessments (hematologic tests and serum biochemistry) and CT or MRI scans.

### 2.4 Evaluation of safety

Safety was monitored during each cycle as standard clinical practice. Lab tests were carried out routinely and laboratory abnormalities were collected. National Cancer Institute Common Terminology Criteria for Adverse Events (CTCAE, version 5.0) were used to grade the AEs.

### 2.5 Evaluation of HRQoL

HRQoL was evaluated using the European Organization for the Research and Treatment of Cancer Quality of Life Questionnaire Core 30 (EORTC QLQ‐C30) (version 3.0).^19^ The QLQ‐C30 comprises five functional scales (physical, role, emotional, cognitive, and social), and nine symptom scales/items (fatigue, nausea and vomiting, pain, dyspnea, insomnia, appetite loss, constipation, diarrhea, and financial difficulties). The score of every item ranged from 0 to 100.^20^ Higher scores on the functional scales indicate higher levels of functioning and better HRQoL, while higher scores on the symptom scales indicate worse symptoms. The questionnaire was administered when a patient had PD or at the last follow‐up.

### 2.6 Statistical analysis

The data cut‐off for this analysis was November 15th, 2022. Data for demographics, clinical outcomes, and AEs were collected retrospectively from patient medical records. For the analysis of effectiveness, patients were excluded if they discontinued eribulin prior to their first radiologic evaluation of tumor response.

Categorical parameters were demonstrated as number (percentage). Differences between groups were evaluated using a chi‐square test or Mann–Whitney nonparametric test where appropriate. Univariable logistic regression analyses were performed to identify potential prognostic factors for median PFS using a Cox proportional hazards model. The risk factors associated with PFS were determined primarily by univariable logistic regression. The characteristics with *p* < 0.05 in the univariable logistic regression analysis were considered as candidates for the multivariable logistic analysis. PFS and OS were estimated by the Kaplan–Meier method and the difference between distinct groups was compared using the log‐rank test. HRQoL was compared for patients who received eribulin monotherapy versus those who received combination therapy. The data were shown as mean value ± standard deviation (SD). Differences between groups (*p* value) were evaluated using the Mann–Whitney nonparametric test.

Data were analyzed using SPSS version 15.0 (SPSS Inc., Chicago, IL). A *p*‐value <0.05 (two‐sided) was considered statistically significant.

## 3 RESULTS

### 3.1 Patient characteristics

Data from 118 patients who had received eribulin to treat MBC, either as monotherapy or as part of combination therapy, were included in the analysis. Patient demographics and baseline clinical characteristics are summarized in Table 1. The median patient age was 50.0 years (range 25–75 years). MBC was HR+ in 69 (58.5%) patients, HER2+ in 18 (15.3%) patients, HR+/HER2− in 61 (51.7%) patients, and 39 patients (33.1%) had triple negative breast cancer (TNBC). Eighty‐three (70.3%) patients had MBC with visceral metastases, in which 48 (40.7%) patients had liver metastasis and 11 (9.3%) patients had brain metastasis. In total, 29 patients had 1 metastatic site before receiving eribulin, 42 patients had 2 metastatic sites, and 47 patients had ≥3 metastatic sites. Eribulin was used in third‐line or later to treat MBC in 78.0% patients, and in second‐line in 22.0% of patients. There was no significant difference in the clinical characteristics of the patients between the eribulin monotherapy and eribulin‐based combination therapy group so that these two groups had sufficiently similar baseline features to allow comparison of clinical outcomes.

**TABLE 1:** Demographic and baseline clinical characteristics of patients with metastatic breast cancer who received eribulin (*N* = 118).

Characteristics	*n* (%)
Age, years [median (range)]	50.0 (25–75)
ECOG performance status score	
0	85 (72.0)
1	22 (18.6)
2	11 (9.3)
Histologic type	
Invasive ductal carcinoma	110 (93.2)
Invasive lobular carcinoma	4 (3.4)
Micro papillary carcinoma	2 (1.7)
Others	2 (1.7)
HR status	
HR+	69 (58.5)
HR–	49 (41.5)
HER2 status	
HER2+	18 (15.3)
HER2–	100 (84.7)
Subtypes	
HR+/HER2–	61 (51.7)
TNBC	39 (33.1)
Metastatic sites	
Lymph nodes	71 (60.2)
Bone	55 (46.6)
Lung	53 (44.9)
Liver	48 (40.7)
Chest wall	30 (25.4)
Brain	11 (9.3)
Adrenal gland	3 (2.5)
Ovary	1 (0.8)
Number of metastatic sites	
<3	71 (60.2)
≥3	47 (39.8)
Current line of treatment for metastatic disease	
2	26 (22.0)
3	29 (24.6)
≥4	63 (53.4)

Abbreviations: ECOG, Eastern Cooperative Oncology Group; HER2, human epidermal growth factor receptor‐2; HR, hormone receptor; TNBC, triple negative breast cancer.

Five patients were excluded because they discontinued eribulin prior to their first radiologic evaluation of tumor response. The average treatment duration for the included patients was 3.6 months and 5.1 therapy cycles were completed by these patients on average.

### 3.2 Tumor response

Tumor responses are summarized in Table 2. While no patient had a CR, 16 (13.6%) had a confirmed PR, resulting in an ORR of 13.6% (16/118). In addition, 73 (61.9%) patients had a SD, resulting in a DCR of 75.4% (89/118). PD was reported in 29 (24.6%) patients. The ORR was 13.0% (7/54) in patients who received eribulin monotherapy and 12.5% (8/64) in patients who received eribulin combination therapy, without significant difference (*p* = 0.940).

**TABLE 2:** Effectiveness of eribulin in patients with metastatic breast cancer (*N* = 118).

Effectiveness measures	Objective response rate, %	Disease control rate, %	Partial response, *n* (%)	Stable disease, *n* (%)	Progressive disease, *n* (%)
All patients (*N* = 118)	13.6	75.4	16 (13.6)	73 (61.9)	29 (24.6)
Line of therapy					
Second line (*n* = 26)	15.4	80.8	4 (15.4)	17 (65.4)	5 (19.2)
Third line (*n* = 29)	10.3	75.9	3 (10.3)	19 (65.5)	7 (24.1)
Fourth‐line or later (*n* = 63)	14.3	73.0	9 (14.3)	37 (58.7)	17 (27.0)
Cancer subtype					
HR+/HER2– (*n* = 61)	13.1	70.5	8 (13.1)	35 (57.4)	18 (29.5)
HER2+ (*n* = 18)	11.1	83.3	2 (11.1)	13 (72.2)	3 (16.7)
TNBC (*n* = 39)	15.4	79.5	6 (15.4)	25 (64.1)	8 (20.5)

Abbreviations: CI, confidence interval; HER2, human epidermal growth factor receptor‐2; HR, hormone receptor; PFS, progression‐free survival; TNBC, triple negative breast cancer.

Eribulin‐based therapy led to a moderate DCR of >70% irrespective of whether it was given in second‐line or later and irrespective of MBC subtype (HR+/HER2–, HER2+, TNBC) (Table 2).

### 3.3 Survival

**FIGURE 1:**
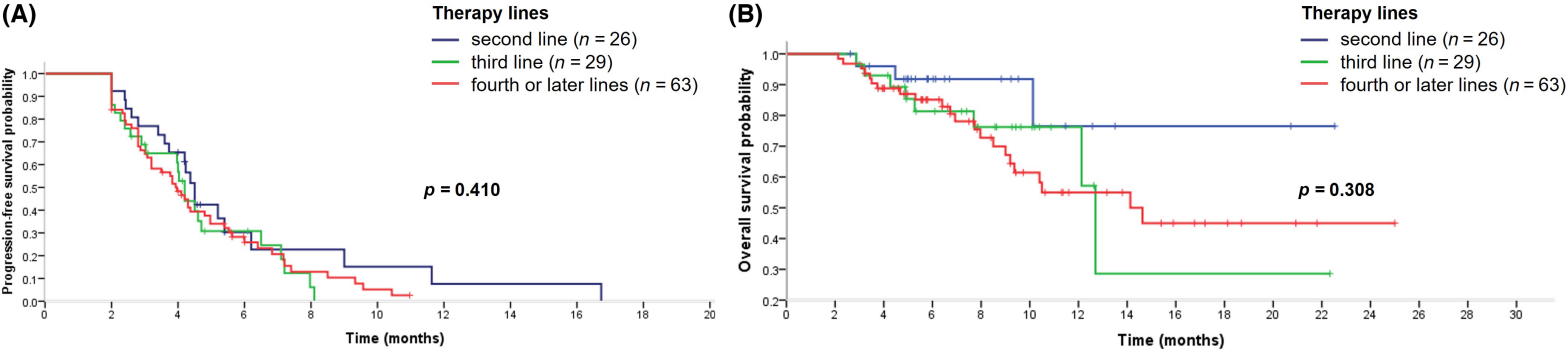
Progression‐free survival and overall survival curves of eribulin‐based therapy in different lines in patients with metastatic breast cancer. (A) Progression‐free survival curve of eribulin‐based therapy in different lines in patients with metastatic breast cancer. (B) Overall survival curves of eribulin‐based therapy in different lines in patients with metastatic breast cancer.

The median PFS in heavily pre‐treated MBC patients was 4.2 months (95% confidence interval [CI]: 3.9–4.5) and the median OS had not been reached. At the data cut‐off, 96 (81.4%) patients had PD, and 22 (18.6%) patients were still receiving eribulin; 85 (72.0%) patients were still alive. The median PFS was 4.5 months in the 26 (22.0%) patients receiving eribulin in second‐line, 4.2 months in the 29 (24.6%) patients receiving third‐line treatment, and 3.9 months in 63 (53.4%) patients receiving eribulin in fourth‐line or beyond (Figure 1A). The median OS had not been reached for patients who received eribulin in second‐line (Figure 1B), while the median OS for patients receiving treatment in third‐ or later line (*n* = 92) was 14.1 months (95% CI 11.0–17.3). The median OS in the patients receiving eribulin in third‐line (*n* = 29) was 12.7 months (95% CI 11.8–13.6) and in those receiving it in fourth‐line or later (*n* = 63) was 14.1 months (95% CI 8.7–19.6) (Figure 1B).

### 3.4 Breast cancer subtypes

**FIGURE 2:**
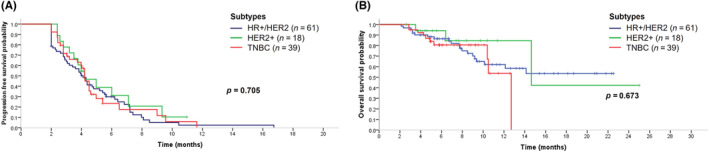
Progression‐free survival and overall survival curves of eribulin therapy in different subtypes of metastatic breast cancer. (A) Progression‐free survival curve of eribulin therapy in different subtypes of metastatic breast cancer. (B) Overall survival curve of eribulin therapy in different subtypes of metastatic breast cancer.

In patients with HR+/HER2– MBC (*n* = 61), the median PFS was 4.1 months (95% CI 3.4–4.8), and was 4.2 months (95% CI 2.8–5.6) in those with HER2+ MBC (*n* = 18), and 4.2 months (95% CI 3.8–4.6) in those with TNBC (*n* = 39). No significant difference was found in PFS among these three groups (*p* = 0.705; Figure 2A). The median OS in patients with HER2+ MBC lesions was 14.6 months (95% CI 3.1–26.2); median OS had not been reached in patients with HR+/HER2− MBC or TNBC (Figure 2B).

### 3.5 Eribulin monotherapy versus combination therapy

**FIGURE 3:**
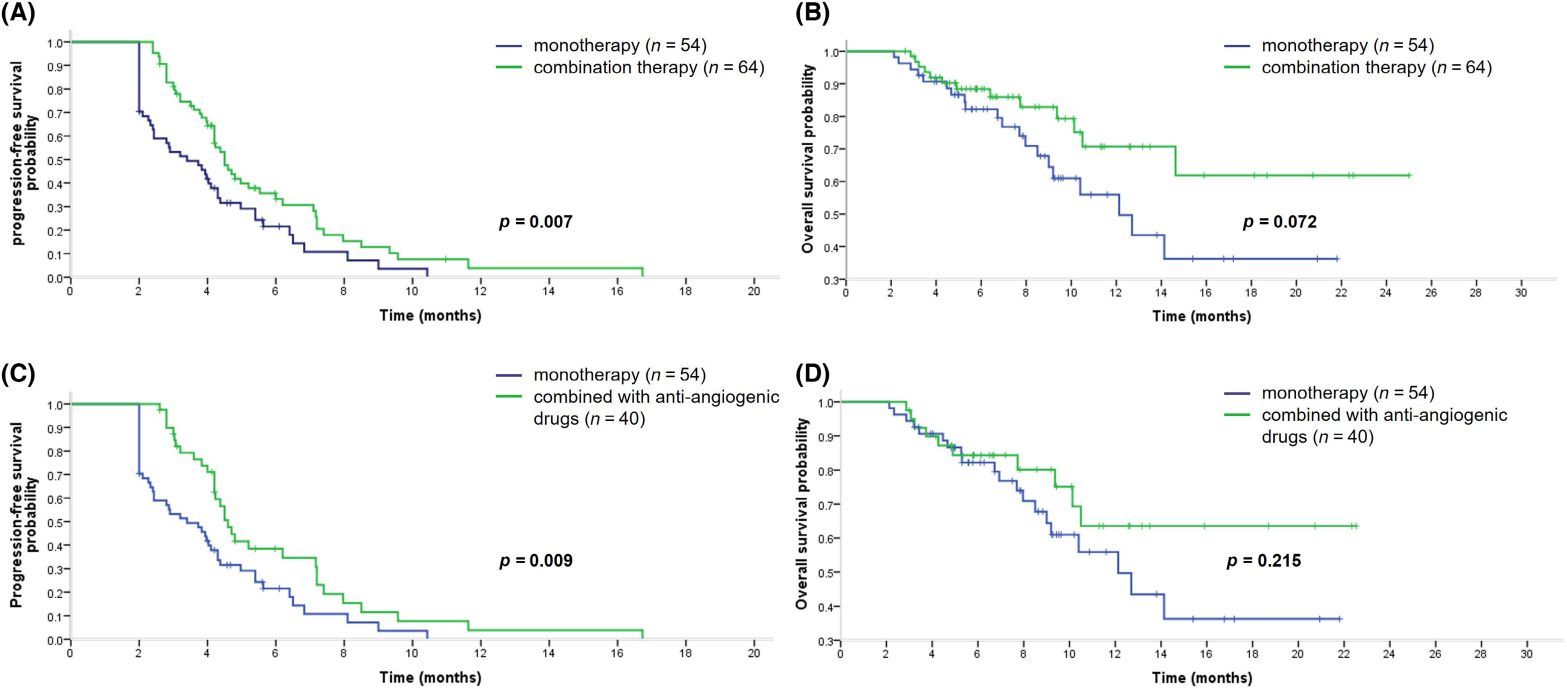
Progression‐free survival and overall survival curves of eribulin monotherapy and combination therapy in patients with anthracyclines and taxanes pre‐treated metastatic breast cancer. (A) Progressionfree survival curve of eribulin monotherapy (*n* = 54) and combination therapy (*n* = 64) in patients with heavily pre‐treated metastatic breast cancer. (B) Overall survival curve of eribulin monotherapy (*n* = 54) and combination therapy (*n* = 64) in patients with heavily pre‐treated metastatic breast cancer. (C) Progressionfree survival curve of eribulin monotherapy (*n* = 54) and eribulin combined with anti‐angiogenic drugs (*n* = 40) in patients with heavily pre‐treated HER2 negative metastatic breast cancer. (D) Overall survival curve of eribulin monotherapy (*n* = 54) and eribulin combined with anti‐angiogenic drugs (*n* = 40) in patients with heavily pre‐treated HER2 negative metastatic breast cancer.

Of the 118 patients, 54 (45.8%) received eribulin monotherapy and 64 (54.2%) received eribulin combination therapy (Table 3). Median PFS was significantly longer in patients who received eribulin combination therapy (4.5 months, 95% CI 4.0–5.0) than in those who received eribulin monotherapy (3.4 months, 95% CI 2.2–4.6; *p* = 0.007, hazard ratio 0.581; Figure 3A). There was a trend towards a longer median OS with eribulin combination therapy (median not reached) compared with eribulin monotherapy (12.1 months, 95% CI 8.6–15.7) but this did not reach statistical significance (*p* = 0.072, hazard ratio 0.531; Figure 3B).

**TABLE 3:** Eribulin monotherapy or combination therapy (*N* = 118).

	*n* (%)
Eribulin monotherapy	54 (45.8)
Eribulin combination therapy	64 (54.2)
Eribulin plus anti‐angiogenic drugs	40 (62.5)
Bevacizumab	28 (23.7)
Anlotinib	9 (7.6)
Apatinib	3 (2.5)
Eribulin plus anti‐HER2 drugs	17 (14.4)
Trastuzumab ± pertuzumab	13 (11.0)
Pyrotinib	3 (2.5)
Neratinib	1 (0.8)
Eribulin plus anti‐PD1 drugs	5 (7.8)
Pembrolizumab	2 (1.7)
Sindilizumab	3 (2.5)
Eribulin plus chemotherapy	2 (1.7)

Abbreviations: HER2, Human epidermal growth factor receptor‐2; PD1, programmed cell death protein 1.

Eribulin was most commonly used in combination with anti‐angiogenic drugs (40 patients, 62.5%): bevacizumab in 28 patients, anlotinib in 9 patients, and apatinib in three patients. The median PFS in patients who received eribulin in combination with anti‐angiogenic drugs (*n* = 40; 4.6 months, 95% CI 4.1–5.1), was significantly longer than in those with HER2– MBC who received eribulin monotherapy (*n* = 54; 3.4 months, 95% CI 2.2–4.6; *p* = 0.009, hazard ratio 0.550; Figure 3C). Median OS was not reached in patients with HER2– MBC who received eribulin combination therapy and was 12.1 months (95% CI 8.6–15.7) in those who received eribulin monotherapy (*p* = 0.215, hazard ratio 0.621; Figure 3D). In 28 patients combined with bevacizumab, the median PFS was 4.5 months (95% CI 3.9–5.1 months).

Of the 18 patients with HER2+ MBC, 17 received eribulin in combination with anti‐HER2 drugs: trastuzumab monotherapy or combined with pertuzumab in 13 patients, pyrotinib in three patients, and neratinib in one patient. The median PFS in patients receiving eribulin in combination with anti‐HER2 therapy was 4.2 months (95% CI 3.4–5.0), with no differences among the anti‐HER2 drugs used (*p* = 0.911).

### 3.6 Predictors of PFS

The only independent factor predicting median PFS in patients with MBC receiving eribulin was the choice of eribulin monotherapy or eribulin combination treatment (hazard ratio 1.721; 95% CI 1.144–2.589; *p* = 0.009; Table 4).

**TABLE 4:** Univariable analysis of the independent predictive factors of median progression free survival of patients with heavily pre‐treated metastatic breast cancer receiving eribulin treatment (*n* = 118).

Factors	Univariable cox	
	HR (95% CI)	*p* value
Age group (<60 vs. ≥60)	0.842 (0.447–1.584)	0.593
ECOG (0–1 vs. 2)	0.867 (0.435–1.728)	0.686
HR status (HR+ vs. HR–)	1.087 (0.718–1.646)	0.694
HER2 status (HER2+ vs. HER2–)	0.790 (0.439–1.421)	0.431
Presence of visceral metastases (yes vs. no)	0.772 (0.500–1.194)	0.245
Number of metastatic sites (<3 vs. ≥3)	1.076 (0.714–1.620)	0.727
Line of therapy (eribulin)		
Second line	1	1
Third line	0.725 (0.424–1.241)	0.241
Fourth‐line or later	1.062 (0.648–1.739)	0.812
Eribulin monotherapy or combination therapy	1.721 (1.144–2.589)	**0.009**

*Note*: Bold print indicates statistical significance.

Abbreviations: CI, confidence interval; ECOG, Eastern Cooperative Oncology Group; HER2, human epidermal growth factor receptor‐2; HR, hazard ratio; HR, hormone receptor.

### 3.7 Safety

Eribulin‐based treatment was generally well tolerated. Table 5 summarizes the AEs reported during the study. The most common grade 3–4 toxicities were neutropenia (22.9%), leukocytopenia (13.6%), and asthenia/fatigue (8.5%). There were no significant differences in the incidences of AEs between the eribulin monotherapy and combination therapy groups. AEs led to dose reductions in 18 (15.3%) patients, specifically grade 3–4 neutropenia/leukocytopenia or asthenia/fatigue. Granulocyte colony‐stimulating factor was used in 35 (29.7%) patients. No episodes of febrile neutropenia or symptomatic cardiac events were reported.

**TABLE 5:** Adverse events in patients who received eribulin for the treatment of metastatic breast cancer (*N* = 118).

	All patients (*N* = 118)	Eribulin monotherapy (*n* = 54)	Eribulin combination therapy (*n* = 64)
Neutropenia	77 (65.3)	35 (64.8)	42 (65.6)
Grades 1–2	50 (42.4)	22 (40.7)	28 (43.8)
Grades 3–4	27 (22.9)	13 (24.1)	14 (21.9)
Leukocytopenia	85 (72.0)	37 (68.5)	48 (75.0)
Grades 1–2	69 (58.5)	31 (57.4)	38 (59.4)
Grades 3–4	16 (13.6)	6 (11.1)	10 (15.6)
Alopecia	72 (61.0)	27 (50.0)	45 (70.3)
Grades 1–2	68 (57.6)	25 (46.3)	43 (67.2)
Grades 3–4	4 (3.4)	2 (3.7)	2 (3.1)
Gastrointestinal toxicities	71 (60.2)	27 (50.0)	44 (68.8)
Grades 1–2	70 (59.3)	26 (48.1)	44 (68.8)
Grades 3–4	1 (0.8)	1 (1.9)	0
Mucositis	71 (60.2)	27 (50.0)	44 (68.8)
Grades 1–2	68 (57.6)	24 (44.4)	44 (68.8)
Grades 3–4	3 (2.5)	3 (5.6)	0
Peripheral neuropathy	71 (60.2)	27 (50.0)	44 (68.8)
Grades 1–2	68 (57.6)	26 (48.1)	42 (65.6)
Grades 3–4	3 (2.5)	1 (1.9)	2 (3.1)
Asthenia/Fatigue	70 (59.3)	27 (50.0)	43 (67.2)
Grades 1–2	60 (50.8)	21 (38.9)	39 (60.9)
Grades 3–4	10 (8.5)	6 (11.1)	4 (6.3)
Elevated transaminases	57 (48.3)	25 (46.3)	32 (50.0)
Grades 1–2	55 (46.6)	24 (44.4)	31 (48.4)
Grades 3–4	2 (1.7)	1 (1.9)	1 (1.6)
Anemia	25 (21.2)	11 (20.4)	14 (21.9)
Grades 1–2	25 (21.2)	11 (20.4)	14 (21.9)
Grades 3–4	0	0	0
Thrombocytopenia	15 (12.7)	7 (13.0)	8 (12.5)
Grades 1–2	13 (11.0)	6 (11.1)	7 (10.9)
Grades 3–4	2 (1.7)	1 (1.9)	1 (1.6)

*Note*: Data are *n* (%).

Abbreviation: CTCAE, National Cancer Institute Common Terminology Criteria for Adverse Events.

### 3.8 HRQoL

The EORTC QLQ‐C30 assessment was completed by 66 of 118 participants (55.9%), among whom 27 patients received eribulin monotherapy and 39 patients received eribulin‐based combination therapy. The proportion of patients with PD in the two groups was 74.1% and 71.8%, respectively. The results were summarized in Table 6. The scores for the nine symptom scales/items for all patients were low. Generally, HRQoL was not significantly different between eribulin combination therapy and monotherapy, although interestingly, cognitive function and symptoms of nausea and vomiting were better with eribulin combination therapy than monotherapy (*p* = 0.034 and *p* = 0.013 vs. monotherapy, respectively).

**TABLE 6:** The quality of life of eribulin‐based therapy in patients with metastatic breast cancer (using EORTC QLQ‐C30 questionnaires).

EORTC QLQ‐C30	All patients (*n* = 66)	Eribulin monotherapy (*n* = 27)	Eribulin combination therapy (*n* = 39)	*p* value
Functional scales				
Physical	82.6 ± 11.3	81.5 ± 8.6	83.3 ± 12.9	0.519
Role	67.2 ± 16.1	64.4 ± 16.5	69.2 ± 15.7	0.228
Emotional	89.8 ± 8.9	89.1 ± 8.6	90.2 ± 9.2	0.623
Cognitive	81.3 ± 14.1	76.9 ± 12.4	84.3 ± 14.5	**0.034**
Social	66.7 ± 13.0	65.3 ± 14.4	67.6 ± 12.1	0.476
Symptom scales/items				
Fatigue	21.6 ± 16.3	24.1 ± 19.4	19.9 ± 13.7	0.306
Nausea and vomiting	10.2 ± 11.4	14.4 ± 12.4	7.4 ± 9.8	**0.013**
Pain	18.9 ± 14.4	20.8 ± 14.7	17.6 ± 14.3	0.379
Dyspnea	10.2 ± 16.4	10.2 ± 17.3	10.3 ± 15.9	0.986
Insomnia	20.5 ± 18.6	23.1 ± 16.9	18.6 ± 19.6	0.33
Appetite loss	15.9 ± 14.3	17.6 ± 16.7	14.7 ± 12.5	0.43
Constipation	5.3 ± 12.0	6.5 ± 11.2	4.5 ± 12.7	0.512
Diarrhea	12.9 ± 15.3	12.0 ± 16.1	13.5 ± 15.0	0.714
Financial difficulties	36.7 ± 16.5	37.0 ± 16.1	36.5 ± 17.1	0.905

*Note*: Data are shown as mean ± SD. Bold print indicates statistical significance. Higher scores in the functional scales represent a superior level of functioning and better health‐related quality of life, while higher scores in the symptom scales/items represent worse symptoms.

Abbreviation: EORTC QLQ‐C30: European Organization for the Research and Treatment of Cancer Quality of Life Questionnaire Core 30.

## 4 DISCUSSION

When eribulin was launched in China in 2019, it was generally used as monotherapy in third‐ or later‐lines of therapy. Since then, eribulin has been investigated in combination with various targeted drugs including anti‐angiogenic agents, anti‐HER2 agents and immune checkpoint inhibitors and other chemotherapeutic agents.^21^, ^22^ In this real‐world study, we demonstrated the effectiveness and safety of eribulin‐based therapy in heavily pre‐treated Chinese patients and provided data on the impact on QoL of eribulin‐based therapy.

In our study, the median PFS was 4.2 months and the median OS in patients who received eribulin monotherapy was 12.1 months. These results are comparable to the phase III trials of eribulin monotherapy (EMBRACE, Study 301, and Study 304), which reported no clear OS benefit despite moderate improvements in PFS (median PFS: 2.8–4.1 months).^9^, ^10^, ^11^ In addition, recent multi‐center, real‐world studies of eribulin in patients with MBC reported median PFS (3.2–6.1 months) and OS (10.1–11.3 months) values in the same range as the present study.^23^, ^24^, ^25^, ^26^, ^27^ Furthermore, we found no significant differences in clinical outcomes across different lines of therapy for MBC or between breast cancer subtypes (HR+/HER2–, HER2+, and TNBC). Interestingly, a systematic review and meta‐analysis of eribulin in locally advanced or MBC found that eribulin in third‐line or beyond was associated with a significant OS benefit versus non‐eribulin therapy, but the benefit only approached significance in first/second line,^28^ which indicated that further evidence from large randomized trials was required to full assess eribulin versus paclitaxel as first‐ or second‐line therapy. In summary, our results further strengthen the evidence that eribulin‐based therapy is an effective treatment option for heavily pre‐treated patients with MBC.

In a recent advance in treatment for MBC, antibody‐drug conjugates (ADCs) have been approved for certain MBC subtypes after exhibiting better effectiveness compared to conventional chemotherapy. For example, both sacituzumab govitecan (SG) and trastuzumab‐deruxtecan (T‐DXd) showed superior outcomes compared to treatment of physician's choice (TPC) in which eribulin was included.^29^, ^30^ However, it should be mentioned that there is limited access to these ADCs in many countries in South America, Eastern Europe, and Asia. In comparison, the results from our study demonstrated a median OS of 14.1 months with eribulin‐based therapy in third‐line or later, which was similar to the OS reported for SG (14.4 months) in the TROPICS‐02 trial.^31^, ^32^ Therefore, eribulin‐based therapy remains an effective treatment option for patients with MBC who have been pre‐treated with anthracyclines and taxanes.

In this study, combining eribulin with other agents appeared to provide additional survival benefits over eribulin monotherapy, particularly when combined with anti‐angiogenic therapies. Eribulin combination therapy was associated with a significantly longer PFS (4.5 vs. 3.4 months) and a trend towards longer median OS compared with eribulin monotherapy. In addition, univariable logistic regression analysis identified eribulin combination treatment as an independent predictor of median PFS. In particular, among patients with HER2− MBC, eribulin plus anti‐angiogenic therapy (most commonly bevacizumab [70%]) led to significantly longer median PFS compared with eribulin monotherapy (4.6 vs. 3.4 months). This finding is consistent with the GIM11‐BERGI trial, in which eribulin plus bevacizumab in second‐line treatment for HER2− MBC led to a median PFS of 6.2 months.^33^ The proposed mechanism behind the clinical benefits of combining eribulin with bevacizumab are that, in addition to eribulin's primary antimitotic mechanism of action, it also triggers a shift from mesenchymal to epithelial phenotypes and induces anti‐vascular activity, inducing tumor vascular remodeling and tumor phenotypic changes that improve the tumor microenvironment.^34^, ^35^

In our study, eribulin‐based therapy was generally well tolerated and the most frequently reported grade 3–4 AEs were neutropenia (22.9%), leukocytopenia (13.6%), and asthenia/fatigue (8.5%). This safety profile is consistent with previous studies of eribulin monotherapy in MBC patients.^9^, ^10^, ^11^ Importantly, grade ≥ 3 symptomatic AEs in our study were relatively rare and included fatigue (8.5%), peripheral neuropathy (2.5%), mucositis (2.5%), alopecia (3.4%) and gastrointestinal toxicities (nausea/vomiting) (0.8%), all of which may be expected to have less of a negative effect on QoL compared with other chemotherapies. For example, other novel microtubule dynamics inhibitors such as nab‐paclitaxel or epothilone are associated with a higher incidence of symptomatic AEs, particularly peripheral neuropathy: 12% with nab‐paclitaxel and 6–24% for epothilone vs. 2.5% in the present study.^36^, ^37^ Peripheral neuropathy results in pain and limb numbness and seriously deteriorates QoL. Furthermore, AEs such as pain and fatigue are more common with taxane‐based therapy compared with eribulin and can seriously affect patient QoL both physically and emotionally.^38^, ^39^ Our results also show that combining eribulin with other agents did not significantly increase the incidence of AEs compared with eribulin monotherapy.

HRQoL is an important factor influencing treatment selection and OS in patients with MBC and AEs related to chemotherapy can greatly reduce HRQoL. Therefore, aiming to prolong OS should be balanced against maintaining HRQoL when treating patients with MBC.^12^, ^40^ As the present study was a retrospective analysis, we were not able to measure changes in HRQoL during eribulin treatment, but we were able to show that the HRQoL associated with eribulin combination therapy was better than eribulin monotherapy in the domains of cognitive function and symptoms of nausea and vomiting. We hypothesize this may be due to the greater effectiveness of combination therapy and better management of AEs during combination therapy. In the phase III trials of eribulin in MBC, there was a low incidence of serious AEs, therefore leading to enhanced QoL.^9^, ^10^ Our results therefore demonstrated eribulin combination therapy might improve PFS and HRQoL without increasing the incidence of AEs.

The present study had several limitations. Recently, several studies have reported promising outcomes for eribulin combined with trastuzumab and pertuzumab in patients with HER2+ MBC or combined with anti‐angiogenesis agents and immune checkpoint inhibitors in HER2− MBC in front‐line therapy.^41^, ^42^, ^43^ Unfortunately, patients included in this study were mainly heavily pre‐treated or had refractory disease, so the effectiveness of eribulin combination therapy in first‐line was not investigated. In addition, as the effectiveness of eribulin based therapy was analyzed retrospectively, there may have been selection bias. The sample size was also relatively small, since eribulin was only available in China since 2019. The effectiveness of different eribulin‐based therapy combinations should be confirmed in a study with a large sample size, particularly for certain combination agents such as bevacizumab. Finally, a longer follow‐up is required to determine the OS.

## 5 CONCLUSIONS

In conclusion, eribulin based therapy was effective and well tolerated in heavily pre‐treated patients with MBC. Eribulin combination therapy might improve PFS and HRQoL compared with eribulin monotherapy. Further large‐scale, prospective clinical trials are required to elucidate the therapeutic effect and safety of specific eribulin combination therapies, such as combinations with immunotherapy and new targeted therapies, in patients with MBC.

## AUTHOR CONTRIBUTIONS

**Xinyu Gui:** Conceptualization (equal); data curation (equal); formal analysis (equal); resources (equal); writing – original draft (equal); writing – review and editing (equal). **Xu Liang:** Conceptualization (equal); data curation (equal); formal analysis (equal); resources (equal); writing – original draft (equal); writing – review and editing (equal). **Huiping Li:** Conceptualization (equal); formal analysis (equal); project administration (equal); resources (equal); writing – review and editing (equal).

## FUNDING INFORMATION

No funding was received.

## CONFLICT OF INTEREST STATEMENT

All the authors declare that they have no competing interests.

## ETHICS APPROVAL AND CONSENT TO PARTICIPATE

The study was approved by the ethics committee of Peking University Cancer Hospital & Institute review board with the approval number of 2020KT75.

## PATIENT CONSENT FOR PUBLICATION

Not applicable.

## Data Availability

All datasets used and/or analyzed are available from the corresponding author on reasonable request.
